# A cMet Inhibitor Promotes the Survival and Long‐Term Efficacy of Transplanted Hepatic Stem/Progenitor Cells in Liver Cirrhosis

**DOI:** 10.1155/sci/7020341

**Published:** 2026-10-30

**Authors:** Min Yan, Deyu Hu, Jiejuan Lai, Quanyu Chen, Jiayu Yu, Mei Ding, Lu Li, Hongbo Shi, Hongyu Zhang, Yu Chen, Lianhua Bai

**Affiliations:** ^1^ Department of Special Medicine Shanxi Medical University Taiyuan City 030001 Sanxi Province China sxmu.edu.cn; ^2^ College of Bioengineering Chongqing University Chongqing City 400044 China cqu.edu.cn; ^3^ Hepatobiliary Institute Southwest Hospital Army Medical University Chongqing City 400038 China tmmu.edu.cn; ^4^ Beijing Key Laboratory Research on Liver Regeneration and Artificial Liver Transplantation Beijing You’an Hospital Capital Medical University Beijing City 100069 China ccmu.edu.cn; ^5^ Department of Hepatobiliary Surgery Chongqing Hygeia Hospital Chongqing City 401147 China

**Keywords:** apoptosis, cirrhosis, cMet inhibitor, HGF, regeneration, stem cells

## Abstract

**Background:**

Poor retention of transplanted stem cells in the liver cirrhosis milieu (LCM) which critically blunts long‐term efficacy, remains unexplained. This study investigated the effects of a small peptide chemical compound (icMet) delivered intravenously for cMet receptor inhibition at the diethylnitrosamine (DEN)‐induced middle‐to‐late cirrhotic liver stage (CLS_8/9_), focusing on apoptosis prevention, endogenous hepatic regeneration and the long‐term efficacy of transplanted hepatic stem/progenitor cells (MLpvNG2 cells).

**Methods:**

C57BL/6 mice with CLS_8/9_ liver injury were used as recipients. icMet was used before or during MLpvNG2 cell therapy. Tissues from CLS_8/9_ livers were also used to generate conditioned medium (CM, CLS_8/9_CM) to observe MLpvNG2 cell activity in response to the injured liver‐related cues. Results: Compared with CLS_8/9_ mice without icMet, mice subjected to icMet administration showed increased survival rates of transplanted MLpvNG2 cells in the CLS_8/9_ livers, as well as enhanced downstream hepatic repair and long‐term healing. In CLS_8/9_CM cultures, the survival of MLpvNG2 cells within icMet was similar to that observed in CLS_8/9_ livers. In terms of the underlying mechanisms, it is possible that icMet pretreatment promotes proliferation and prevents apoptosis of grafted MLpvNG2 cells, activating host hepatic functional differentiation, particularly sinusoidal reconstruction, which can initiate hepatocyte regeneration. In addition, the relieved fibrotic load could also favor repopulation by donor cells, leading to long‐term treatment efficacy.

**Conclusions:**

A strategy involving a pre‐ or during cell treatment that blocks the cMet receptor achieves apoptosis resistance of transplanted MLpvNG2 cells in the LCM and promotes endogenous hepatic regeneration, leading to long‐term treatment efficacy.

## 1. Introduction

Liver cirrhosis (LC) is a consequence of chronic liver injury. Pathologically, this disease is characterized by excessive scarring with fibrosis, circumscribing parenchymal nodules [1]. Numerous factors, such as viral infection, nonalcoholic or alcoholic fatty liver disease, and drug abuse, can cause fibrosis [2–4]. Subsequently, fibrotic tissues can replace the normal parenchyma, resulting in a remodeled tissue architecture and, ultimately, compromised liver function. At present, the only therapeutic option for LC is orthotopic liver transplantation. However, this treatment is tremendously limited because of the shortage of donor organs. Therefore, alternative treatment options are urgently needed. Stem cell therapy, such as treatment with mesenchymal stem cells (MSCs) [5] or hepatic stem/progenitor cells (HSPs) [6], is a promising option for bridging organ shortages and has emerged as a novel alternative for treating this disease.

Our previous studies have identified that MLpvNG2 cells are HSPs and exhibit MSC‐like features [7]. Transplanting MLpvNG2 cells into a mouse model of diethylnitrosamine (DEN)‐induced early stage of cirrhotic liver injury, a well‐accepted animal model [8], has been shown to exert strong therapeutic effects through direct hepatic functional differentiation, immune response modulation, and liver‐protective cytokine production [9]. However, although obvious improvement of liver injury has been achieved by stem cell or HSP transplantation [10], unresolved challenges still limit therapeutic outcomes. Among these limitations, determining the primary cause of massive cell loss in the harsh middle‐to‐late liver cirrhosis milieu (LCM), which impacts long‐term therapeutic effectiveness after cell transplantation, has become increasingly crucial issues. For example, our previous study revealed that in the livers of DEN‐treated mice, most endogenous HSPs (eNG2 cells) survive in the inflammatory DEN‐induced fibrotic liver stage, 3–6 weeks after DEN administration (FLS_3–6_ post‐DEN), but the cells undergo massive apoptosis in the cirrhotic liver stage, 7–10 weeks after DEN administration (CLS_7–10_ post‐DEN) [11], and the mechanism remains unclear.

A widely accepted theory is that the majority of stem cells die after transplantation when they encounter pathological stresses beyond their maximal tolerance in the adverse milieu and enter into an irreversible cell death program or apoptosis rather than stably colonizing the tissue to exert therapeutic effects [12, 13]. Thus, strategies to promote the survival of transplanted stem cells in the LCM after transplantation are critical for optimizing the outcomes of stem cell‐based therapies for LC. In the present study, we describe a strategy involving inhibition of the cMet receptor with a small peptide chemical compound (icMet) at the DEN‐induced middle‐to‐late stage of LC (CLS_8/9_) before MLpvNG2‐HSP transplantation and report how this approach protects the grafted cells from death by apoptosis in response to CLS_8/9_ cues and initiates endogenous hepatic regeneration to promote long‐term treatment efficacy. A possible underlying mechanism is also interpreted.

## 2. Materials and Methods

### 2.1. Animals

Male C57BL/6 mice (aged 8–10 weeks, 22 ± 2 g) were purchased from Army Medical University (Chongqing, China). All of the animals were housed in a specific pathogen‐free environment and received human care according to protocols approved by the University Animal Care Committee (#SYXK‐PLA‐2012‐00120031).

### 2.2. DEN‐Induced Liver Fibrosis/Cirrhosis Mouse Model and Treatment Groups

Through normal drinking water, the mice received a small amount of DEN (99.5% purity, 22 ± 2 g) at a concentration of 0.01% (v/v)/0.014% (0.13 mg/mL, 25.86 mg/kg) daily for 6 days and at 1‐day intervals beginning on the 7th day for 12 consecutive weeks. The DEN‐induced fibrotic/cirrhotic chronic liver injury mouse model effectively mimics the pathological progression of LC observed in humans, progressing from normal tissue to fibrosis, cirrhosis, and cancer [11]. The fibrotic liver stage occurred 3–6 weeks after the administration of DEN (FLS_3–6_, post‐DEN), the CLS occurred 7–10 weeks (CLS7‐10) post‐DEN, and cancer developed up to 10 weeks post‐DEN; fibrosis development of these pathological processes was determined by Masson’s trichrome staining (blue) [14]. In this study, tissues were collected from FLS_3/4_ and CLS_8/9_ livers and were designated as fibrotic stage and middle‐to‐late stage tissues, respectively. DEN‐treated mice were randomly assigned to three groups: (1) the DEN‐induced groups, which included the FLS_3/4_ and CLS_8/9_ subgroups; (2) the MLpvNG2 cell therapy groups, which included subgroups subjected to the CLS_8/9_ mice treated with MLpvNG2 cells for 3 days (NG2‐3d) and 4 weeks (NG2‐4wk); and (3) the icMet groups, which received icMet intravenously at CLS_8/9_ stage 18–24 h before transplantation with MLpvNG2 cells for 3 days (icMet + NG2‐3d, assumed short‐term therapy) and during transplantation with MLpvNG2 cells (3 shots) for 4 weeks (icMet + NG2‐4wk, assumed long‐term therapy) (Figure S1B, blue arrows). The control mice were made to drink normal water. At least 15 mice were present in each group. The animals were sacrificed at 3 days and 4 weeks after the MLpvNG2 cell transplantation via the tail vein to assess the short‐ and long‐term efficacy, respectively. The mortality rate of mice ranged from 10% to 15%.

### 2.3. Purification of MLpvNG2 Cells From the Adult Mouse Liver, Cell Labeling, and Experimental Design

MLpvNG2 cells were isolated using the Percoll‐Plate‐Wait procedure (PPWP), as described in our previous study [7]. Briefly, liver tissues around the portal vein were obtained from 6‐ to 8‐week‐old mice, and the tissue blocks were placed in modified Eagle’s medium without calcium and magnesium ions (_W_MEM) and chilled. These blocks were subsequently dissociated in digestive solution for 30 min and centrifuged at 800×*g* for 5 min. The cell suspension was thoroughly rinsed and placed on a Percoll gradient. Afterward, the sample was centrifuged at 2000 rpm for 30 min, and the cellular fraction was carefully collected, washed, and resuspended in 1 mL of DMEM/F‐12 supplemented with 10% fetal bovine serum (FBS) (complete medium). Cells were then seeded into a 75 cm^2^ culture flask at a density of 1 × 10^5^ cells/mL/set. The cells were maintained at 37°C in 5% CO_2_ for at least 3 weeks, after which 1–3 mL of complete medium was added once per week. After 3 weeks, the medium was changed every 3 days until the cells reached 80% confluence. The cells were passaged at least once.

The purified MLpvNG2 cells were labeled with CellTracker, either carboxyfluorescein diacetate succinimidyl ester (CFSE) or RedCM‐DiI (RedDye), before they were used for transplantation. The cells were injected into DEN model mice via the tail vein (1 × 10^5^ cells/mouse in 200 µL) and analyzed at 18, 24, 72 h, and 4 weeks. The treatment groups continued to receive DEN and were monitored for an additional 3 days or 4 weeks. After the animals were sacrificed by cervical dislocation, liver samples from each animal were collected and processed as follows: (a) fixed in 4% buffered formaldehyde and embedded in paraffin, (b) snap frozen at −80°C for sectioning and immunohistochemistry or RNA isolation, and (c) homogenized in appropriate buffer(s) for preparing conditioned medium (CM) and biochemical assays.

### 2.4. CM Preparation and Coculture With MLpvNG2 Cells

The types of CM included (1) control medium (Ctrl‐CM), which was composed of DMEM/F12 supplemented with 10% FBS; (2) FLS_3/4_ and CLS_8/9_ liver homogenates mixed with Ctrl‐CM without FBS (FLS_3/4_CM, CLS_8/9_CM) at a 1:4 or 1:8 v/v after centrifugation at 170 × *g* for 10 min and passage through a 0.45‐μm filter; and (3) culture of MLpvNG2 cells for 18–24 h in CLS_8/9_CM (CLS_8/9_CM + NG2) or the CLS_8/9_CM 24 h before cells treatment with icMet (100 ng/mL [15], icMet‐CLS_8/9_CM + NG2). Additionally, four doses of the recombinant hepatocyte growth factor (rHGF) protein (1, 5, 150, and 300 ng/mL) were used for MLpvNG2 cell culture compared to the cultures without rHGF (0 ng/mL), and proliferation and apoptosis were assessed through immunofluorescence (IF) staining for Ki‐67 and terminal deoxynucleotidyl transferase (TdT) dUTP nick‐end labeling (TUNEL) staining, respectively.

### 2.5. IF and Immunohistochemical (IHC) Staining

For IF staining of cells grown on coverslips, MLpvNG2 cells were plated at a density of 1 × 10^4^ cells per coverslip, treated with different types of CM, and cultured for 18–72 h. Primary antibodies against the following proteins were used: Ki‐67; albumin (ALB) and glucose‐6‐phosphatase (G6Pc), which are markers of mature hepatocytes; cytokeratin 19 [CK19, a marker of bile duct cells (BDCs)]; alpha‐smooth muscle actin (α‐SMA), a marker of activated hepatic stellate cells (HSCs); lymphatic vessel endothelial hyaluronan receptor‐1 (LYVE‐1), a liver sinusoidal endothelial cell (LSEC)‐specific marker; and Fas, cMet, caspase‐8 and phosphorylated tyrosine (pTyr). All the antibodies were diluted 1:100 in DMEM supplemented with 5% normal goat serum (NGS). After being incubated overnight at 4°C in a humidified chamber, the cells were washed 6 times for 5 min with DMEM. Goat antirabbit secondary antibodies conjugated to Alexa Fluor 488 (green), Alexa Fluor 594, or Cy3 (red) were incubated with the cells at a 1:400 dilution at room temperature (RT) for 1 h. The cells were rinsed 6 times with PBS and mounted using Vectashield containing 4^′^‐6‐diamidino‐2‐phenylindole (DAPI). The proportion of positive cells was determined in 3 randomly selected areas per coverslip across at least three independent experiments by using 3 independent stem cell preparations. The data were assessed using independent *t*‐tests. For IHC staining of liver sections, mice were perfused with 4% paraformaldehyde (PFA). Liver tissues were cryoprotected in 30% sucrose overnight, embedded in optimal cutting temperature (OCT) compound, and sectioned at 5 μm with a Leica cryostat (Leica CM1950, Wetzlar, Germany). The sections were stained with diaminobenzidine (DAB, brown) to detect HGF and neuron–glia antigen 2 (NG2). Positive areas or cells were identified as brown‐stained regions in the tissue sections. Tissues from different animals (*n* = 6) were assayed, with at least 16 sections obtained for each animal. All stained cells were imaged under a fluorescence microscope (Olympus BX53F2), and liver sections were imaged with a phase‐contrast microscope (Olympus BX41). All images were captured by an investigator who was blinded to the treatments administered to individual animals.

### 2.6. Apoptosis Assays

Apoptosis was estimated in both cultured cells and frozen liver sections using a TUNEL assay kit according to the manufacturer’s instructions. The cells were fixed in 5% acid methanol (−20°C) for 12 min and washed 2 times for 5 min in RT DMEM supplemented with 5% NGS. The 5‐μm sections were fixed with 4% PFA and stained with the reagents provided with the kit. Five to 10 sections from each mouse were randomly selected, and three mice were used per treatment group. Stained cells with nuclear labeling were considered TUNEL‐positive apoptotic cells and were visualized using a Nikon ECLIPSE Ti series fluorescence microscope at 400× magnification (Tokyo, Japan).

### 2.7. Migration Assays

To determine the effects of MLpvNG2 cell homing to DEN‐treated livers at either FLS_3/4_ or CLS_8/9_ in vivo, RedDye‐labeled MLpvNG2 cells were allowed to migrate for 18–24 h after transplantation. The liver tissues were fixed with cold methanol, and the labeled cells were traced. To evaluate cell invasion in vitro, the 200 μL cells at a density of 2 × 10^5^ were seeded into the upper chamber of a 24‐well Transwell system (Boyden chamber assay) [16] precoated with Matrigel and cultured in serum‐free medium in the upper compartment. FLS_3/4_CM and CLS_8/9_CM were added to the lower compartment, respectively, and compared the cells cultured in Ctrl‐CM. After 18–24 h, the cells in the chambers were fixed with 4% PFA and stained with crystal violet. Cell migration was quantified by counting the cells that passed through the filter. Stained cells from a minimum of five fields of view for three replicates were counted, and the data are presented as the average number of migrated cells. For the wound‐healing assay [16], cells were seeded in a 6‐well plate and grown until they reached 80% confluence. A sterile plastic tip was used to create a scratch in the cell layer before CM was added. After 48 h, images were captured with a microscope, and the migration distance was quantified as the number of wound closures. Migration was monitored using a phase‐contrast microscope (Olympus BX53F2) to assess cell invasion and wound healing.

### 2.8. Coimmunoprecipitation (Co‐IP)

Co‐IP was performed to detect cMet–Fas interactions in healthy MLpvNG2 cells, as described in a previous study [17]. The cells were lysed in Co‐IP buffer (20 mM Tris, pH 7.5; 150 mM NaCl; 1% Triton X‐100; and 1 mM EDTA) containing protease inhibitors on ice for 30 min. Then, the supernatant was collected and sequentially incubated with primary antibodies against cMet or Fas and GammaBind Plus Sepharose with gentle rocking overnight at 4°C, and the mixture was subsequently pelleted, washed 6 times with cold 1× Co‐IP buffer, and analyzed through immunoblotting.

### 2.9. Immunoblotting

Proteins extracted from liver tissues and cultured MLpvNG2 cells were separated on a 10% sodium dodecyl sulfate (SDS)‐polyacrylamide gel, transferred to polyvinylidene difluoride membranes, and incubated with monoclonal primary antibodies against HGF, Fas, Fas‐associated protein death domain (FADD), caspase‐8, and caspase‐3. Horseradish peroxidase (HRP)‐conjugated secondary antibodies were incubated with the membranes, and the bands were visualized with an enhanced chemiluminescence system (ECL, GE Healthcare Biosciences, Piscataway, NJ) according to the manufacturer’s instructions.

### 2.10. Quantitative Reverse Transcription‐PCR (qRT‐PCR) Analysis

Total RNA was purified from liver tissues and cultured stem cells using TRIzol reagent. A reverse transcriptase RT‐PCR kit was used to reverse transcribe RNA (2 μg) and obtain cDNA. The RNA concentration was quantified using a NanoDrop 8000 spectrophotometer. PCR was performed using a 7500 real‐time PCR detection system (Thermo Fisher Scientific) The PCR cycling conditions used were as follows: 40 cycles of 95°C for 5 s and 60°C for 30 s. The sequences of primers targeting *hgf*, *krt19*, *alb*, *g6Pc*, *lyve1, and acta2* (Sangon Biotech, Shanghai, China) are presented in Table S1. The expression of target mRNAs was normalized to that of *actb*, which served as an endogenous control. Relative changes in expression were determined using the 2^*−ΔΔCt*^ method.

### 2.11. Analysis of Endogenous Hepatic Regeneration

After different treatments, functional hepatic differentiation in mice with DEN‐induced liver fibrosis/cirrhosis was detected through IF staining with antibodies against CK19, LYVE‐1, ALB, and G6Pc.

### 2.12. Measurement of Serum Levels of Hepatic Functional Markers

Blood samples were collected from the retroorbital plexus of mice [18] anesthetized with isoflurane by using a glass capillary and centrifuged at 10,000 rpm for 10 min. Serum samples were prepared as previously reported [7], subsequently aliquoted, and stored at −80°C. The serum levels of ALB, alanine transaminase (ALT), aspartate aminotransferase (AST), low‐density lipoprotein (LDL), total bilirubin (TBiL), and direct bilirubin (DBiL) in the different subgroups were measured using a Beckman Count Chemistry Analyzer (AU5800).

All the primary and secondary antibodies, reagents, kits, and buffers used in the study are listed in Table S2.

### 2.13. Human Sample Preparation

Human tissue samples were obtained from the Biobank of the Department of Pathology at the Army Medical University (Chongqing, China). The study utilized archived formalin‐fixed paraffin‐embedded (FFPE) or frozen sections in accordance with the university’s standard protocols and ethical guidelines (Approval No. KY2025122). The inclusion criteria required a histologically confirmed diagnosis of HBV‐related cirrhosis or hemangioma with nonspecific local inflammation (*n* = 3 per type), as well as the availability of sufficient high‐quality tissue for immunofluorescent staining. Samples with excessive necrosis or insufficient material were excluded.

### 2.14. Statistical Analysis

The data are presented as the means ± SDs. Statistical analyses of data from two groups were performed using an unpaired Student’s *t* test (two‐tailed) or analysis of variance (ANOVA). One‐way ANOVA followed by Dunnett’s test was used to compare the values obtained for multiple groups. All experiments were repeated at least 3 times, and *p* < 0.05 was considered to indicate statistical significance. Also, when multiple measurements or technical replicates were obtained from each biological sample, all statistical analyses and significance tests were strictly based on the number of independent biological replicates (n).

## 3. Results

### 3.1. Differences in the Survival of Transplanted MLpvNG2 Cells Administered After Different Durations of DEN‐Induced Liver Injury

The administration of DEN resulted in the development of chronic liver injury (Figure S1A), from a naive state to fibrosis (FLS_3–6_), cirrhosis (CLS_7–10_), and cancer up to 10 weeks post‐DEN (pink arrow); these pathological processes were confirmed through Masson’s trichrome staining (blue). The overall design of the MLpvNG2 cell treatment (black arrows) for DEN‐induced liver injury and analysis at 3 days and 4 weeks after transplantation is shown in Figure S1B. The survival of endogenous HPCs (eNG2 cells) during the FLS_3–4_ period was significantly greater (Figure S1C, left two panels, arrows) than that observed during the CLS_8–9_ period (Figure S1C, right two panels, arrows), as determined by IF staining with an antibody against neuroglia antigen 2 (NG2, red). Compared with those in the CLS_8–9_ period, more Ki‐67^+^ cells (green) were costained with NG2^+^ in the FLS_3–4_ period (Figure S1D, merged, arrows). By contrast, double IF plus TUNEL staining unveiled the opposite results: fewer TUNEL^+^ cells (green) were costained with NG2^+^ in the FLS_3–4_ period than in the CLS_8–9_ period (Figure S1E, merged, arrows) [11].

Herein, we selected the FLS_3/4_ and CLS_8/9_ periods to represent fibrosis and the middle‐to‐late stage of LC, respectively, andr transplanted MLpvNG2 cells, respectively, into the two periods. MLpvNG2 cells were isolated using the PPWP (*Mat/Met*) and show 95%–98% purity after ex vivo expansion, as determined by IF staining for NG2 (red, Figure 1A) with HSP properties [9]. The number of grafted RedDye‐labeled MLpvNG2 cells was shown significantly lower in the CLS_8/9_ livers (Figure 1B2, arrow) than the cells in the FLS_3/4_ livers (Figure 1B1, arrows, Figure 1B3) 18–24 h after transplantation. IF staining revealed that fewer than 33.02% ± 12.79% of MLpvNG2^+^ cells (RedDye) were positive for Ki‐67 (green) in the CLS_8/9_ livers (Figure 1C, lower panels, merged, arrow), in contrast to the higher percentage of merged cells in the FLS_3/4_ livers (67.46% ± 16.02%, Figure 1C, top panels, arrows), The number of grafted MLpvNG2 cells significantly decreased during the CLS_8/9_ period (*p* < 0.001; Figure 1D1), and the decrease was approximately two and a half times (−2.5‐fold) less than that observed in the FLS_3/4_ period (Figure 1D2). IF staining also revealed that approximately ⁓77% of the MLpvNG2 cells (RedDye) in the CLS_8/9_ livers were TUNEL‐positive (green, Figure 1E2, merged, arrows), while this percentage was only ⁓12% in the FLS_3/4_ livers (Figure 1E1, merged, arrow). Further analysis revealed significant differences in TUNEL staining between the two group cells (Figure 1F, *p* < 0.001). These findings suggest that the transplanted MLpvNG2 cells survived during the inflammatory FLS_3/4_ period but exhibited poor retention during the CLS_8/9_ period. These observations indicate that the fate of MLpvNG2 cells is similar to that of endogenous HSPs (eNG2 cells) [9] after transplantation into the LCM.

**Figure 1:**
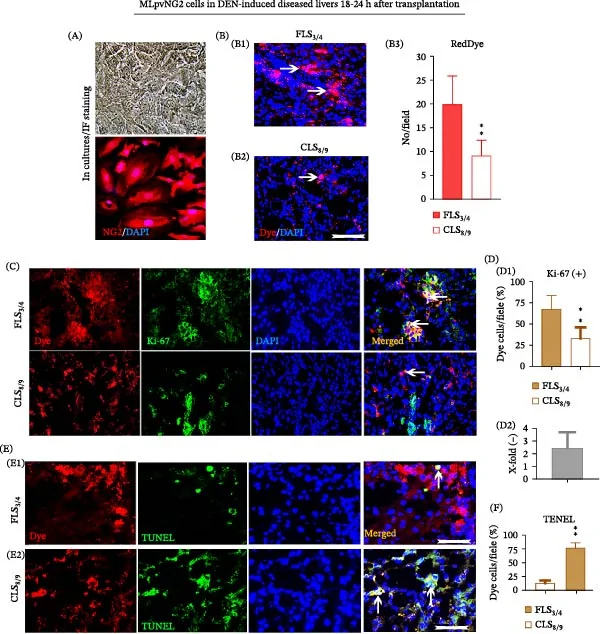
Differences in survival among transplanted MLpvNG2 cells during different periods of DEN‐induced liver injury. (A) MLpvNG2 cells in normal cultures and cultures subjected to IF staining for the NG2 protein (NG2, red). (B1–B3) RedDye‐labeled MLpvNG2 cells in FLS_3/4_ (B1, arrows) and CLS_8/9_ (B2, arrow) livers 18–24 h after transplantation and quantification of the number of migrating cells (B3); *n* = 3. (C) IF staining for Ki‐67 (green) in RedDye‐labeled MLpvNG2 cells in FLS_3/4_ (top panels) and CLS_8/9_ (low panels) livers 18–24 h after transplantation. (D1, D2) Quantification of the data in C (D1) and fold changes derived from the data in C (D2, CLS_8/9_/FLS_3/4_); *n* = 6 mice/group. (E1, E2) TUNEL staining (green) of RedDye‐labeled MLpvNG2 cells in FLS_3/4_ (E1) and CLS_8/9_ (E2) livers. (F) Quantification of the data in E1 and E2 (merged images, arrows); *n* = 6 mice/group. The term “field” refers to randomly selected areas within tissue sections from each biological replicate (*n*), the statistical analyses were performed based on the number of biological replicates (*n* = 6). The results are presented as the means ± SDs of at least three independent experiments. Scale bar = 200 μm in all the images.  ^∗∗^ *p* < 0.001 compared with FLS_3/4_.

### 3.2. Differences in the Migration of MlpvNG2 Cells Toward Cues From the DEN‐Induced FLS_3/4_ and CLS_8/9_ Liver

To determine whether MlpvNG2 cell viability can be influenced by different liver injury cues, the cells were cultured in liver‐generated CMs (FLS_3/4_CM and CLS_8/9_CM) to observe their growth and potential of responding to injured cues. In the FLS_3/4_CM cultures, the cells exhibited greater colony formation ability (Figure 2A2, pink arrows) than those in the Ctrl‐CM cultures (Figure 2A1). By contrast, more apoptotic‐like cells (Figure 2A3, white arrows) and significantly fewer colony‐forming cells were observed in the CLS_8/9_CM cultures than in the FLS_3/4_CM cultures (Figure 2B) after 18–24 h of culture, suggesting poor retention of MLpvNG2 cells under cirrhotic conditions.

**Figure 2:**
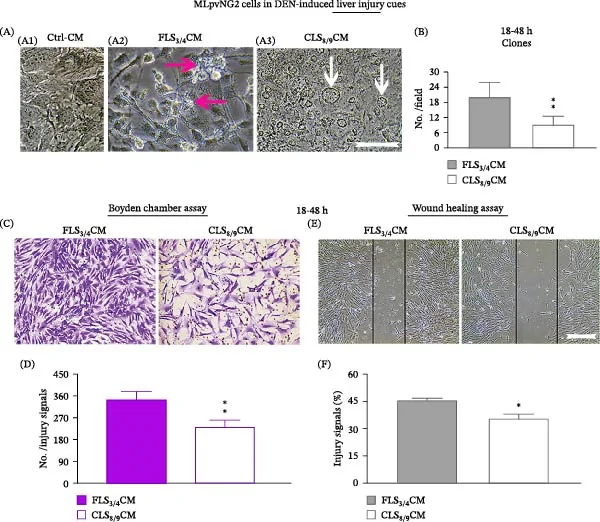
Differences in the ability of MlpvNG2 cells to migrate toward cues from the DEN‐induced FLS_3/4_ and CLS_8/9_ liver. (A1–A3) MLpvNG2 cells in Ctrl‐CM (A1), FLS_3/4_CM (A2) and CLS_8/9_CM (A3) subcultures after 18–24 h; pink arrows indicate proliferative cell clones, and white arrows indicate apoptotic‐like cells; three livers/subgroup. (B) Quantification of the numbers of MLpvNG2 cell clones from the A2 and A3 subgroups. (C, D) MLpvNG2 cells were exposed to FLS_3/4_CM and CLS_8/9_CM for 18–24 h in a Boyden microchemotaxis chamber to identify potential chemoattractants, and cells that had migrated toward the source of the chemoattractant (lower chamber) by passing through a membrane filter (8 μm) were visualized by crystal violet staining (C) and quantified (D), with three sets/experiment. (E, F) In the wound‐healing assay, confluent monolayers of MLpvNG2 cells were scratched with a pipette tip. After 24–48 h in FLS_3/4_CM and CLS_8/9_CM cultures, wound closure was documented photographically (E) and quantified (F). The figures show one representative result out of five. Bars = 200 μm in the images in A and 100 μm in the images in C.  ^∗^ *p* < 0.05 and  ^∗∗^ *p* < 0.001 compared with FLS_3/4_CM.

By performing an in vitro study using a Boyden chamber assay, we confirmed that after 18–24 h cultures, a significant lower number of pvNG2 cells in the CLS_8/9_CM cultures than in FLS_3/4_CM cultures (Figure 2C,D, *p* < 0.001). The results of the wound‐healing assay showed similar trend to those observed in the Boyden chamber. After 24–48 h of culture, the MlpvNG2 cells in FLS_3/4_CM could heal part of the wound area, resulting in a significantly (*p* < 0.05) more efficient healing process than the cells cultured with CLS_8/9_CM (Figure 2E,F). MlpvNG2 cells cultured in Ctrl‐CM were used as a positive control, and the control cells filled the original wound area (not shown). These findings suggest that signals from FLS_3/4_ livers are more beneficial for MlpvNG2 cell retention, whereas signals from CLS_8/9_ livers are harmful for cell retention. These observations indicate that MlpvNG2 cells may not be able to tolerate the harsh cirrhotic microenvironment and therefore undergo death via apoptosis.

### 3.3. HGF Levels are Higher in the DEN‐Induced Cirrhosis Period Than in the Inflammatory Fibrosis Period

The mechanisms through which different periods of liver injury elicit opposing effects on HPC (eNG2 [11] and MLpvNG2) survival and apoptosis remain unexplained. Given that HGF affects cell activity in a dose‐dependent manner under physiological conditions [19] and high HGF levels have been observed in the injured liver of pathological condition [20], we hypothesized that high HGF levels in cirrhotic livers may also induce MLpvNG2 cell apoptosis after transplantation. IHC (DAB brown) staining revealed that HGF expression was indeed significantly (*p* < 0.05) greater in CLS_8/9_ livers than in FLS_3/4_ livers (Figure 3A, boxes and Figure 3B). Western blotting (Figure 3C, n1–n4 represent individuals) and qRT‐PCR (Figure 3D) revealed similar trends. Notably, the IHC staining results also revealed that HGF levels were significantly negatively correlated with endogenous HPCs (eNG2, markered as NG2) in CLS_8/9_ livers (same sections) (Figure 3E,F, *R* ^2^ = 0.7850, *p* < 0.0001). These findings suggest that the poor retention of MLpvNG2 cells in the cirrhotic niche could be due to a strong burst of HGF stress. Interestingly, a similar pattern was seen in human liver samples with cirrhosis (hcirLivers, Figure S2B) where double IF staining identified a significant (*p* < 0.001) less number of NG2^+^ cells (red, h/NG2 denoted as box) compared to same staining in the nonspecific inflammatory hemangioma (HA) liver samples (Figure S2A, box and Figure S2C, *n* = 3). Furthermore, we detected a dramatic high TUNEL expression (green) costained with NG2 (red, h/NG2) in the hcirLivers (Figure S2B, merged, arrows) compared to the staining in HA (Figure S2A, merged, arrow). The percentage of TUNEL^+^ cells (green) costained for hNG2 in hcirLivers was significantly greater than that in HA livers (Figure S2D, *p* < 0.001), hinting that there maybe similar HSP cell behavior in hcirLivers to in animals with cirrhosis. Of note, this human study is just for preliminary observation, and the sample size (*n* = 3/type) is not enough to infer general patterns, and the conclusions need to be verified with a larger sample. These observations indicate that excessive HGF in cirrhotic livers may be a major pathological factor that directly induces hepatic stem cell apoptosis, regardless of whether intrahepatic [11] or ex vivo‐expanded extrahepatic stem cells (MLpvNG2) [7] are used.

**Figure 3:**
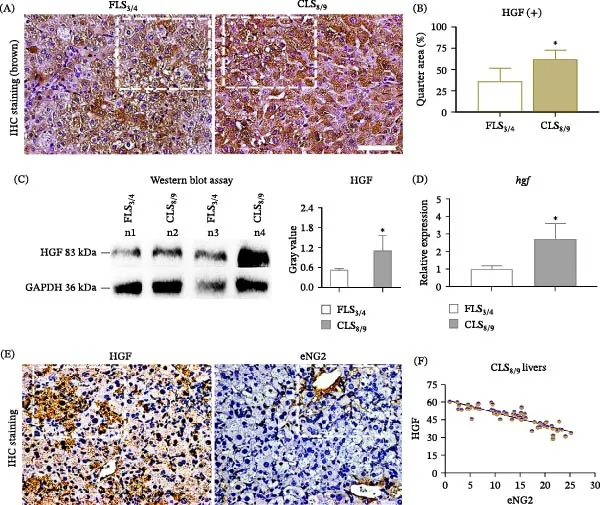
HGF levels are higher in the DEN‐induced cirrhotic period (CLS_8/9_) than in the inflammatory fibrotic period (FLS_3/4_). (A, B) IHC (DAB) staining (brown) for HGF expression in FLS_3/4_ and CLS_8/9_ livers (A, boxes) and quantification (B), the “quarter area” represents aggregated data derived from one‐quarter of the observation fields per replicate and statistical analyses were performed based on the number of biological replicates, *n* = 6/subgroup. (C) HGF expression in FLS_3/4_ and CLS_8/9_ livers was tested by western blot analysis and quantification, *n* = 2/per type of tissue groups (FLS_3/4_, CLS_8/9_). (D) HGF mRNA expression levels in the subgroups detected by a qRT‐PCR assay, *n* = 3. (E, F) IHC staining for the expression of HGF and endogenous HSPs (eNG2) in the same sections from CLS_8/9_ liver tissues (E, *n* = 6 mice; boxes) and quantitative analysis of the negative correlation between the two proteins (F, *R* ^2^ = 0.7850; *p* < 0.0001), each data point represents the aggregate of 8 fields from a single biological replicate. The bars represent 200 μm in all of the images, and the results are presented as the means ± SDs of at least three independent experiments;  ^∗^ *p* < 0.05 compared with FLS_3/4_ livers.

### 3.4. HGF Protein Directly Affects MLpvNG2 Cell Survival in a Dose‐Dependent Manner

To confirm that a strong burst of HGF in the LCM could directly induce MLpvNG2 cells apoptosis, we cultured healthy MLpvNG2 cells directly with different concentrations of rHGF, from low concentrations (1, 5 ng/mL) to high concentrations (150, 300 ng/mL), over 24–72 h. Increased cell growth and clone numbers were observed in the cultures with 1 and 5 ng/mL rHGF (Figure 4A2, pink arrows) compared with the cultures in which rHGF was not added (Figure 4A1), and the cultures treated with 1 or 5 ng/mL rHGF showed significant increases (Figure 4C1,C2,  ^∗^ *p* < 0.05) over those without rHGF (Figure 4C, denoted as scale 0). When up to 150 or 300 ng/mL rHGF was added, obvious apoptotic‐like cells appeared (Figure 4B1,B2, white arrows), and the cell activity decreased significantly (Figure 4C3,C4, #*p* < 0.05). A similar trend was confirmed by IF staining for Ki‐67 in the cultures, which revealed a greater percentage of Ki‐67^+^ MLpvNG2 cells (green) among the cells cultured with 1 or 5 ng/mL rHGF (Figure 4D2, white arrows and Figure 4E1,E2) than among the cells in Ctrl‐CM cultures (Figure 4D1,E, denoted as scale 0). By contrast, the percentage of Ki‐67^+^ MLpvNG2 cells significantly decreased after treatment with 150 or 300 ng/mL rHGF (Figure 4D3,D4,E3,E4). TUNEL staining (green) was substantially increased in the 150 ng/mL rHGF cultures (Figure 4F2, white arrows) compared with cultures without rHGF (Figure 4F1, arrow and Figure 4F3). These findings suggest that grafted MLpvNG2 cells enduring HGF stress in cirrhotic livers might be in a dose‐dependent manner. These observations hint that the dual role of HGF signaling can be observed not only in hepatocytes under pathological conditions [19] but also in hepatic stem cells (eNG2, hNG2, and MLpvNG2 cells) under pathological conditions.

**Figure 4:**
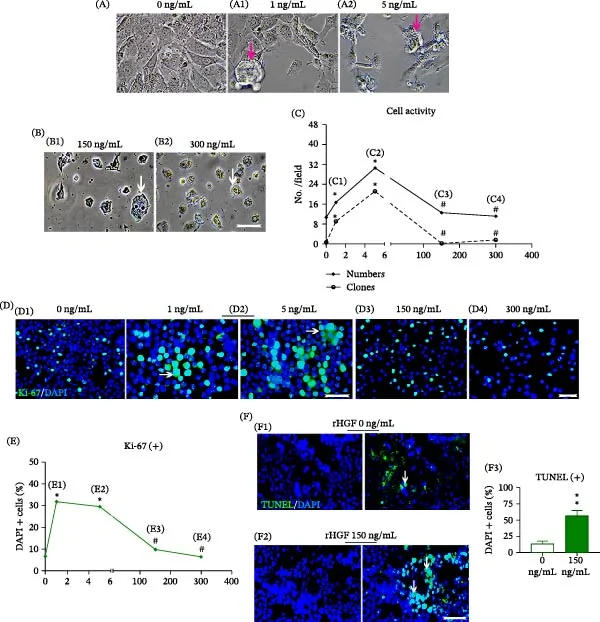
The HGF protein directly affects MLpvNG2 cell survival in a dose‐dependent manner. (A1, A2) MLpvNG2 cells in different cultures with 1 and 5 ng/mL rHGF (A2) or without rHGF (A1); pink arrows indicate clonal cells, and white arrows indicate apoptotic‐like cells. (B1, B2) MLpvNG2 cells in cultures with 150 ng/mL (B1) and 300 ng/mL (B2) rHGF; white arrows indicate apoptotic‐like cells. (C1–C4) Quantification analysis from cultures of MLpvNG2 within low concentration of rHGF 1 ng/mL (A1) and 5 ng/mL (A2) (C1, C2) and high concentration of rHGF 150 ng/mL (B1) and 300 ng/mL (B2) (C3, C4); the solid line represents the number of MLpvNG2 cellswhile the dashed line represents the clone numbers of the cells (C) from A1, A2 and B1, B2. (D1–D4) Ki‐67^+^ MLpvNG2 cells in cultures with rHGF at concentrations of 1 and 5 (D2) or 150 and 300 ng/mL (D3, D4) or without rHGF (D1); *n* = 6/set. (E1–E4) Quantification of the data from D1 and D2 (E1, E2) or from D3 and D4 (E3, E4). (F1–F3) TUNEL staining (green, arrows) of cultured MLpvNG2 cells (denoted as DAPI^+^ cells) in the presence of 150 ng/mL rHGF (F2) or in the absence of rHGF (F1) and quantification of the subgroups (F3); *n* = 6/subgroup. All the data are presented as the means ± SDs of duplicate preparations from three independent (culture) experiment or six independent (tissue) biological replicates *(n)*, the statistical analyses were performed based on the number of biological replicates (*n*). Scale bar = 100 μm in the images in D3 and D4, and scale bar = 200 μm in the other images.  ^∗^#*p* < 0.05 and  ^∗∗^ *p* < 0.001 for the comparison of control (0 ng/mL rHGF) cells and cells treated with 1 or 5 ng/mL rHGF.

### 3.5. Prior Inhibition of HGF Signaling With a cMet Inhibitor Prevents the Apoptosis of Transplanted MLpvNG2 Cells and Promotes Functional Recovery

Considering a previous report that cMet is the only receptor for HGF [21], we next tested whether blocking cMet could improve the retention of MLpvNG2 cells in the LCM. After treating DEN‐induced mice with a cMet inhibitor (icMet) at the cirrhotic stage (CLS_8/9_), we found that by 72 h after transplantation, the injected CFSE‐labeled MLpvNG2 cells (1 × 10^5^/200 μL/animal, IV) were much more likely to attach to the icMet‐treated injured livers (icMet + NG2‐3d, arrows) than to livers that were not treated with icMet (NG2‐3d, arrow) (Figure 5A, *p* < 0.001). Immunostaining for Ki‐67^+^ cells (red, Figure 5B2) among grafted CFSE‐labeled MLpvNG2 cells (denoted as CFSE, Figure 5B2, green) in CLS_8/9_ recipient mice (icMet + NG2‐3d) greatly increased (Figure 5B2, merged, arrows) compared with that observed in the recipient mice treated with MLpvNG2 cells but not subjected to icMet pretreatment (NG2‐3d, Figure 5B1, merged, arrow). More than 29% of the grafted MLpvNG2 cells in the icMet + NG2‐3d recipient animals expressed Ki‐67, while <7% of the grafted MLpvNG2 cells in the NG2‐3d animals expressed Ki‐67, with a significant difference noted between the two groups (Figure 5B3, *p* < 0.001); the observed staining was approximately fourfold greater in the icMet + NG2‐3d group than in the NG2‐3d group (Figure 5B4). Notably, the percentage of Ki‐67^+^ cells among grafted MLpvNG2 cells in the icMet + NG2‐3d group (merged image in Figure 5B5, denoted as a box and Extra in an analyzed bar in Figure 5B7) was much greater (28.72% ± 9.06%) than the endogenous percentage of Ki‐67^+^ cells (7.28% ± 2.69%; Figure 5B6, denoted as a box which merged green and red panels from Figure 5B2, and Intra in Figure 5B7), suggesting a significant (*p* < 0.001) competitive advantage of grafted MLpvNG2 cells over host cells in terms of proliferation may contribute to liver repopulation [22, 23]. Importantly, animals transplanted MLpvNG2 in icMet CLS_8/9_ livers (icMet + NG2‐3d) exhibited significant improvement in serum hepatic functions, as evidenced by reduced levels of liver injury‐related proteins such as ALT, AST, and LDL (Figure 5C1–C3, pink bars), and excretory function‐related TBiL and DBiL (Figure 5C4,C5, blue bars), compared with those in the animals treated with only cells (NG2‐3d). The levels of these liver proteins in DEN‐induced animals indicated marked severe damage such as ALT (Figure 5C1, dark bar) compared with this in normal animals (ALT, Figure 5C1, gray bar). Other items, such as AST, LDL, TBiL, and DBiL, tended to exhibit similar trends in DEN‐treated livers compared to normal livers (not shown). However, although MLpvNG2 cell treatment for 3 days (NG2‐3d) significantly improved ALT levels (Figure 5C1, ##*p* < 0.001), as well as AST, LDL, TBiL and DBiL (not shown) levels; while when the benefical activities in the icMet strategy group (icMet + NG2‐3d) was much better than the NG2‐3d groups, such as ALT (Figure 5C1, *p* < 0.001), along with other markers AST, TBiL and DBiL (Figure 5C2,C4,C5, *p* < 0.05), but the effect of LDL shows not as good as NG2‐3d group (Figure 5C3, *p* < 0.05). IF staining for α‐SMA (red) revealed a decreased number of α‐SMA‐labeled cells in both animal groups within 72 h compared with that in DEN‐treated animals (not shown), but the number of α‐SMA^+^ cells was significantly (*p* < 0.05) lower in the icMet + NG2‐3d group animals (Figure 5D, boxes), suggesting that the antifibrotic activity of grafted MLpvNG2 cells was more effective with the assistance of icMet. To assess host hepatic regeneration in the two groups, liver sections were labeled with antibodies against CK19, a cholangiocyte marker. In the lesion areas, the number of CK19^+^ cells was fourfold greater (26.84 ± 6.64/7.81 ± 3.92) in the icMet + NG2‐3d animals (Figure 5E2, arrows) than in the NG2‐3d animals (Figure 5E1, arrow), suggesting that the biliary tree was significant repaired (Figure 5E3, *p* < 0.001). Importantly, the number of LYVE‐1^+^ cells, a marker of LSECs, was also greatly increased in the icMet + NG2‐3d livers (Figure 5F2, box) compared with the NG2‐3d livers (Figure 5F1, box and Figure 5F3), suggesting that the repaired LSECs structures support hepatocyte regeneration through releasing HGF [24]. These observations indicate that pretreatment with icMet before donor cell transplantation can prevent the apoptosis of MLpvNG2 cells in the LCM after transplantation, enabling the transplanted MLpvNG2 cells to promote biliary tree and sinusoidal structure reconstruction.

**Figure 5:**
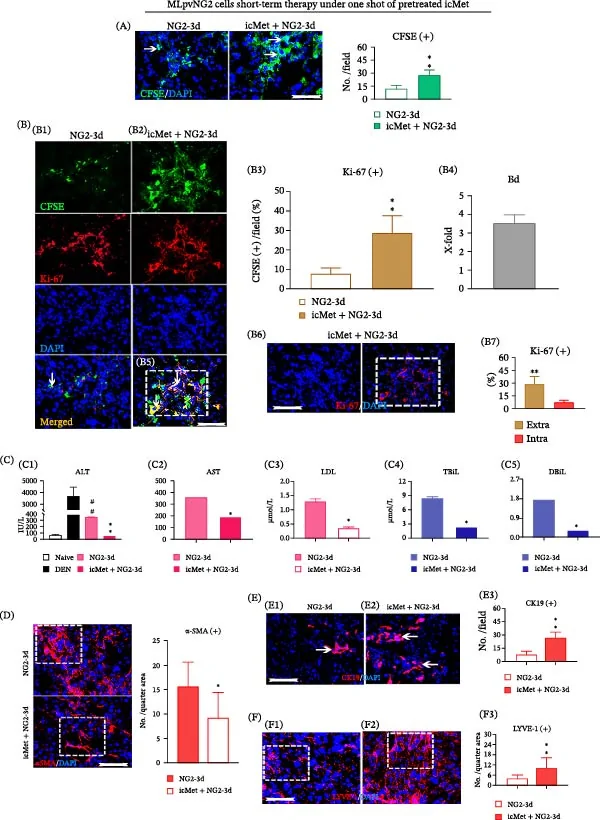
Prior inhibition of HGF signaling with a cMet inhibitor promotes transplanted MLpvNG2 cell proliferation, hepatic repair and functional recovery 3 days after transplantation. (A) The number of CFSE‐labeled MLpvNG2 cells in livers from different subgroups (arrows) and quantitative analysis after the cells were allowed to home to the livers with (icMet + NG2‐3d) or without (NG2‐3d) icMet treatment; *n* = 3/group. (B1–B4) IF staining for Ki‐67 (red) in CFSE‐labeled grafted MLpvNG2 cells in icMet pretreated livers (icMet + NG2‐3d) (B2) and without icMet livers (NG2‐3d) (B1); arrows indicate merged costaining; quantification of the data in B1 and B2 pointed by arrows (B3, *n* = 6) and fold changes (B4) are shown; (B5–B7) Comparison of Ki‐67^+^ cells among MLpvNG2 cells (B5 merged, box, denoted as Extra) with endogenous Ki‐67^+^ cells in B6 (merged from B2 red and blue panels, denoted as Intra), and quantitative analysis (B7), *n* = 6. (C1–C3) Measurement of serum levels of the ALT (C1), AST (C2), and LDL (C3) in the NG2‐3d and icMet + NG2‐3d subgroups and in the DEN‐treated (C1, dark bar, ALT) and naive (C1, gray bar, ALT) livers; (C4, C5) Measurement of serum levels of the hepatic proteins TBiL (C4) and DBiL (C5) in the NG2‐3d and icMet + NG2‐3d subgroups with a Beckman Count Chemistry Analyzer; *n* = 6. (D) IF staining (red) and quantification of host α‐SMA^+^ cells (boxes). (E1–E3) IF staining (red) of CK19^+^ cells in the NG2‐3d (E1, arrow) and icMet‐NG2‐3d (E2, arrows) groups and quantification (E3). (F1–F3) IF staining (red) of LYVE‐1^+^ cells in the NG2‐3d (F1, box) and icMet‐NG2‐3d (F2, box) subgroups and quantification (F3); *n* = 6/marker. The data are representative of triplicate experiments, and counts represent the means ± SDs of at least three experiments; scale bar = 200.  ^∗^ *p* < 0.05 compared with the NG2‐3d group;  ^∗∗^ *p* < 0.001 compared with the NG2‐3d and intra groups.

### 3.6. The icMet Pretreatment Strategy Promotes Hepatocyte Regeneration and Functional Maintenance of MLpvNG2 Cells in a DEN‐Induced Decompensated Cirrhosis Model

Given that the reconstruction of sinusoidal structures (LYVE‐1) suggested the initiation of regeneration [24], the occurrence of such changes should support the therapeutic effects of the donor cells. To test this hypothesis, we next examined hepatocyte regeneration by using G6Pc and ALB markers, comparing the icMet + NG2‐3d group and the MLpvNG2 treatment group for 4 weeks under the icMet treatment strategy (icMet + NG2‐4wk). As expected, the numbers of both G6Pc^+^ (arrows, Figure 6A1) and ALB^+^ (boxes, Figure 6B1) hepatocytes were significantly greater in the icMet + NG2‐4wk group than in the icMet + NG2‐3d group (Figure 6A2,B2), as determined by IF staining (red). Similar mRNA expression trends were observed by qRT‐PCR (Figure 6A3,B3), confirming substantial hepatocyte regeneration. Analysis of hepatic functional proteins in serums with a Beckman Count Chemistry Analyzer revealed that LDL levels continued to increase (Figure 6C2, *p* < 0.05) in icMet + NG2‐4wk group animals compared with icMet + NG2‐3d group animals when both group animals were with icMet pretreatment; the levels of other protein markers, such as ALT (Figure 6C1, *p* = 0.911), AST (Figure 6C3, *p* = 0.65), and TBiL (Figure 6C4, *p* = 0.383), suggesting that with the assistance of icMet, the transplanted MLpvNG2 cells could exert long‐term and maintenance therapeutic effects. Furthermore, when compared the icMet + NG2‐4wk group animals with the animals subjected to the same treatment but without icMet pretreatment (NG2‐4wk), we found that marked decreases in AST (Figure 6D1, *p* < 0.05), TBiL (Figure 6D2, *p* < 0.001) and DBiL (Figure 6D4, *p* < 0.05) but increased LDL levels (Figure 6D3, *p* < 0.001), in the icMet‐treated recipient mice compared to ones in only cell‐treated group (NG2‐4wk), suggesting that prior inhibition of the cMet receptor could not only repair injured hepatocytes obviously (AST) but significantly improve excretion (TBiL, DBiL) and lipid transport (LDL) functions as well. Regarding the LDL showing different directions in Figures 5C3 and 6C2,D3 suggest that this protein requires time to exert its effect. Interestingly, we found that serum ALB protein levels were remarkably improved at 4 weeks after cell transplantation with icMet assistance (icMet + NG2‐4wk) compared with the 3 days treatment (icMet + NG2‐3d) (Figure 6E2), with levels showing no significant difference from the normal control group (Figure 6E3, *p* = 0.111), although we did not see a significant difference in effects between icMet + NG2‐3d group and the group treated with MLpvNG2 cells only at the short‐term timepoint (NG2‐3d) (Figure 6E1, *p* = 0.9), suggesting that ALB‐based hepatocyte regeneration requires a specific time frame. In addition, attenuation of fibrosis was closely correlated with the icMet treatment strategy, as IF staining for α‐SMA (red) revealed that the number of α‐SMA^+^ cells was dramatically lower in the icMet + NG2‐4wk group animals than in the icMet + NG2‐3d group animals (Figure 6F1, boxes, *p* < 0.05). A similar trend was detected for the *abta2 gene* by qRT‐PCR (Figure 6F2, ***p* < 0.05**). These observations indicate that the icMet plus stem cell treatment strategy strongly promotes hepatocyte regeneration, long‐term functional recovery and maintenance, and relieves the fibrotic load.

**Figure 6:**
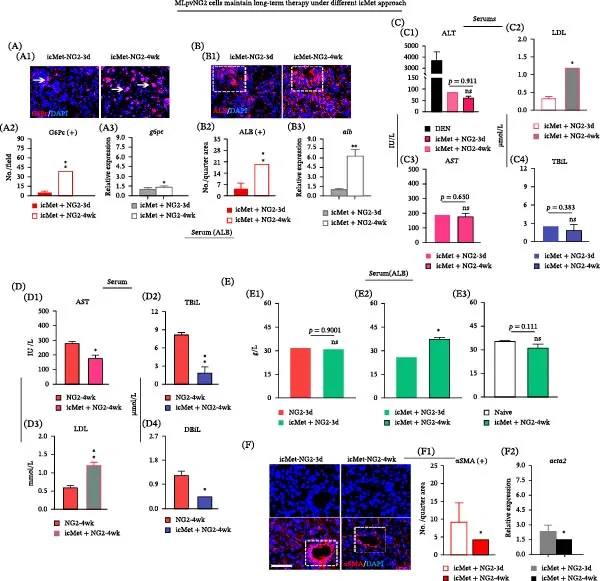
The icMet treatment strategy promotes hepatocyte regeneration and long‐term functional maintenance of MLpvNG2 cells in a DEN‐induced decompensated cirrhosis model. (A1–A3) IF staining (red) for G6Pc (A1, arrows indicate G6Pc^+^ cells), quantification (A2) and of its mRNA levels by qRT–PCR (A3) in the icMet + NG2‐3d and icMet + NG2‐4wk subgroups; *n* = 6/method. (B1–B3) IF staining (red) for ALB (B1, boxes indicate ALB^+^ cell regions), quantification (B2) and of its mRNA levels by qRT–PCR (B3) in the icMet + NG2‐3d and icMet + NG2‐4wk subgroups; *n* = 6/method. (C1–C4) Comparative quantification of ALT (C1), LDL (C2), AST (C3), and TBiL (C4) levels in serum samples between short‐ and long‐term subgroups. (D1–D4) Quantification of AST (D1), TBiL (D2), LDL (D3), and DBiL (D4) in the group treated with MLpvNG2 cells only for 4 weeks (NG2‐4wk) and the group pretreated with icMet plus MLpvNG2 donor cells (icMet + NG2‐4wk). (E1–E3) Quantification of serum ALB levels for the following comparisons: (E1) 3 days of treatment with MLpvNG2 cells in DEN animals (NG2‐3d) compared with icMet + NG2‐3d animals; (E2) icMet + NG2‐3d group compared with icMet + NG2‐4wk group; (E3) naive compared with icMet + NG2‐4wk animals, *n* = 6/group. (F1, F2) IF staining for a‐SMA (red) plus quantification (F1) and *abta2 gene* analysis at the mRNA level (F2) in animals from the icMet + NG2‐3d and icMet + NG2‐4wk subgroups; *n* = 6. The data are from at least three experiments; scale bar = 200.  ^∗^ *p* < 0.05 compared with the 3d and NG2‐4wk groups, and  ^∗∗^ *p* < 0.001 compared with the 3d NG2‐4wk group; ns indicates that the difference was not significant.

### 3.7. Efficient Survival of MLpvNG2 Cells and Host Long‐Term Bile Duct and Sinusoidal Regenerations in the LCM Supported the 4‐Week Observation Period Therapy

With respect to the mechanism of the 4‐week observation period healing mediated by MLpvNG2 cells, we speculated that the effect may be due mainly to the efficient survival of MLpvNG2 cells, which mediate host long‐term hepatic regeneration [9]. To prove this, we compared short‐term (icMet + NG2‐3d) and long‐term (icMet + NG2‐4wk) treatment with CFSE‐labeled MLpvNG2 cells under the cMet treatment strategy. IF staining (red) for Ki‐67 demonstrated a greater percentage of Ki‐67^+^ cells in icMet + NG2‐4wk livers than in icMet + NG2‐3d livers (Figure 7A1, merged, arrows), and this difference was statistically significant (Figure 7A2, *p* < 0.001). Conversely, significantly fewer apoptotic TUNEL^+^ (red) MLpvNG2 cells^+^ (CFSE) observed in the icMet pretreated animal livers 4 week after cell transplantation (merged, Figure 7B2, box) than the icMet pretreated animal 3 days after cell transplantation (merged, Figure 7B1, box, Figure 7B3, *p* < 0.001), suggesting that prior to donor cells treated with icMet promotes greater cell retention by stimulating proliferation and preventing apoptosis. IF staining (red) further revealed considerably increased abundance of CK19^+^ cholangiocytes in the livers of the icMet + NG2‐4wk group mice (Figure 7C2, arrows) than in those of the icMet + NG2‐3d group mice (Figure 7C1, arrow; Figure 7E1, *p* < 0.001). Moreover, a similar expression pattern was detected in host LYVE‐1^+^ cells (Figure 7D1,D2, boxes, Figure 7E2, *p* < 0.05). Similar trends in *krt19* and *lyve-1* mRNA expression levels are shown in Figure 7F1 (*p* < 0.05) and Figure 7F2 (*p* < 0.05), respectively, as indicated by qRT‐PCR, suggesting that the ongoing reconstruction of sinusoidal structures facilitating liver regeneration may continuously aid in biliary tree restoration, although the chronological order of these processes remains to be determined. These observations indicate that with the assistance of icMet, MLpvNG2 cells can survive for the 4‐week observation period in the LCM after transplantation, which is beneficial for improving host biliary tree continue reconstruction; in particular, sinusoidal structure reconstruction which supports hepatocyte regeneration initiation (Figure 6A,B,E2,E3) [25–27], thus promoting sustained therapeutic efficacy.

**Figure 7:**
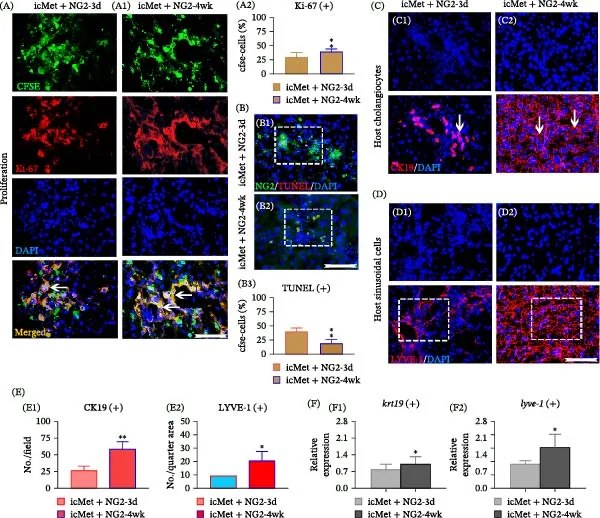
Efficient survival of MLpvNG2 cells and host hepatic regeneration in the LCM support long‐term therapy. (A1, A2) IF staining (red) of Ki‐67^+^ cells among grafted CFSE‐labeled MLpvNG2 cells in the icMet + NG2‐3d and icMet + NG2‐4wk subgroups (A1, merged, arrows) and quantitative analysis of these results (A2); *n* = 6/group. (B1–B3) IF staining (red) of TUNEL^+^ cells among grafted CFSE‐labeled MLpvNG2 cells (merged, boxes) in the icMet + NG2‐3d (B1) and icMet + NG2‐4wk (B2) subgroups and quantitative analysis of the two groups (B3); *n* = 6/group. (C1, C2) Comparative analysis of host CK19^+^ cholangiocytes (arrows) in the icMet + NG2‐3d (C1) and icMet + NG2‐4wk (C2) subgroups by IF staining (red). (D1, D2) Comparative analysis of host LYVE‐1^+^ liver sinusoidal cells (boxes) in the icMet + NG2‐3d (D1) and icMet + NG2‐4wk (D2) subgroups by IF staining (red). (E1, E2) Quantification of the staining data in C (E1) and D (E2). (F1, F2) Quantification of *krt19* (F1) and *lyve-1* (F2) mRNA expression levels in liver tissues from the two groups; *n* = 3/marker. The experimental outcomes are presented as the means ± SDs of three independent experiments. Scale bar = 200 μm in all the images.  ^∗^ *p* < 0.05 and  ^∗∗^ *p* < 0.001 compared with the icMet + NG2‐3d group.

### 3.8. Preinhibition of cMet Prevents Fas‐Mediated Apoptosis and Stimulates Proliferation of MLpvNG2 Cells in Response to Cirrhotic Cues

To understand the mechanism underlying the 4‐week period survival of transplanted MLpvNG2 cells in the icMet‐pretreated LCM, we first performed a coculture experiment. After 18–24 h of culturing the MLpvNG2 cells in CLS_8/9_CM (CLS_8/9_CM + NG2), an obvious apoptotic‐like appearance was observed (Figure 8A2, pink arrows). However, when icMet (100 ng/mL) was added to the cultures 24 h before the cells (icMet‐CLS_8/9_CM + NG2), the cells returned to normal in terms of morphology (Figure 8A3), similar to those in the Ctrl‐CM cultures (Figure 8A1), suggesting that cMet inhibition could prevent MLpvNG2 cell death in the LCM. Given that the cell death in response to cirrhotiliver injury signals, we next performed comparative analyses. Treated MLpvNG2 cells with CLS_8/9_CM in the absence (CLS_8/9_CM + NG2) or presence (icMet‐CLS_8/9_CM + NG2) of icMet (24 h before the cells), we found that after 18–24 h, a significant decrease in Fas expression (Figure 8B1, *p* < 0.05), TUNEL staining (Figure 8B2, *p* < 0.001) and caspase‐8 expression (Figure 8B3, *p* < 0.05) in the icMet‐CLS_8/9_CM + NG2 cultures (Fas: 15.46 ± 5.71; TUNEL: 17.55 ± 5.36; caspase‐8: 2.63 ± 1.61) compared with the CLS_8/9_CM + NG2 cultures (Fas: 24.69 ± 6.03; TUNEL: 37.26 ± 11.62; caspase‐8: 6.88 ± 2.22), determined by IF staining (red, arrows). Similar expression patterns of some apoptotic events FADD, caspase‐8 (denoted as casp‐8), and caspase‐3 (denoted as casp‐3) were shown in western blotting assay between the cells in CLS_8/9_CM only (NG2, Figure 8C2) and the cultures pretreated with icMet (icMet + NG2, Figure 8C1); gray value analysis revealed significant decreases (Figure 8C3, *p* < 0.05, *n* = 6). This finding suggests that the icMet treatment strategy inhibits the ability of MLpvNG2 cells to undergo Fas‐mediated apoptosis. Interestingly, given the dose‐dependent response of the MLpvNG2 cells to the HGF protein (rHGF), with 150–300 ng/mL rHGF causing MLpvNG2 cell apoptosis (Figure 4B1,B2,F2), we confirmed using western blot analysis that the apoptosis induced by high concentrations (200 ng/mL) of the purified HGF protein (rHGF) was mediated by Fas, as the three related death markers showed significantly increased expression (Figure 8D2) compared with that in the cultures without rHGF (0 ng/mL, Figure 8D1,D3, *p* < 0.05, *n* = 6). Given that the cMet–Fas complex [19] naturally exists in healthy MLpvNG2 cells (Figure 8E, by Co‐IP assay), further supporting that the sensitivity of grafted MLpvNG2 cells to death in response to HGF/cirrhotic stress is related to Fas‐mediated apoptosis.

**Figure 8:**
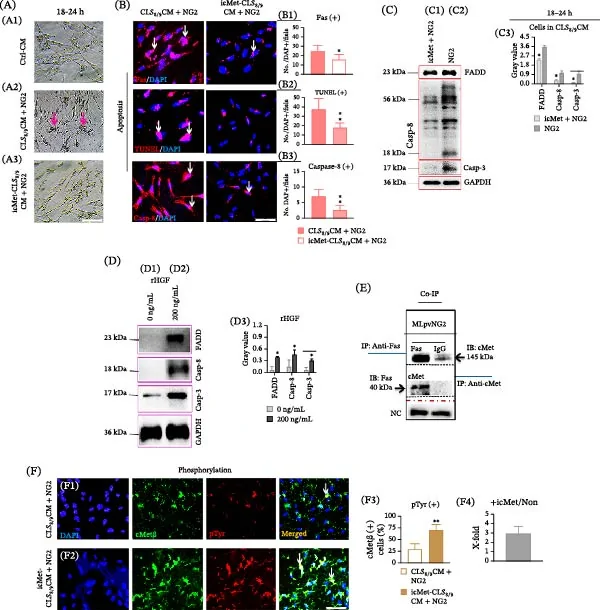
icMet treatment prevents Fas‐mediated apoptosis and stimulates the proliferation of MLpvNG2 cells in the cirrhotic microenvironment. (A1–A3) MLpvNG2 cells were cultured for 18–24 h in normal medium (Ctrl‐CM, A1), in CLS_8/9_CM (CLS_8/9_CM + NG2; A2, pink arrows indicate apoptotic‐like cells) and in CLS_8/9_CM plus icMet 24 h before MLpvNG2 cell addition (icMet‐CLS_8/9_CM + NG2; A3); *n* = 3/set. (B1–B3) IF staining (red) for apoptosis markers (denoted as arrows) in MLpvNG2 cells when the cells were cultured in CLS_8/9_CM or CLS_8/9_CM preplus icMet, and quantitative analysis of the expression of Fas (B1), TUNEL (B2), and caspase‐8 (B3) in MLpvNG2 cells. (C1–C3) Measurement of FADD, caspase‐8 and caspase‐3 expression in MLpvNG2 cells cultured in CLS_8/9_CM (C2) and medium supplemented with icMet (C1) by western blotting and quantitative analysis (C3) after 18–24 h of culture. (D1–D3) Western blot analysis of the expression of FADD, caspase‐8 and caspase‐3 in healthy MLpvNG2 cells in normal cultures for 18–24 h in the absence (D1, 0 ng/mL) or presence (D2, 200 ng/mL) of rHGF and quantitative analysis (D3); *n* = 3/set. (E) Co‐IP confirmed the existence of cMet–Fas complex in healthy MLpvNG2 cells. (F1–F4) Double IF staining to detect tyrosine phosphorylation (pTyr, red)^+^ among cMetβ (green)‐expressing MLpvNG2 cells (DAPI^+^) in CLS_8/9_CM cultures with (F2, merged, arrows) or without (F1, merged, arrow) icMet treatment, quantitative analysis of the data from F1 and F2 (F3) and fold changes from the data in F2/F1 (F4). At least three independent experiments were performed; the data are presented as the means ± SDs. Scale bar = 200 μm. # ^∗^ *p* < 0.05 and  ^∗∗^ *p* < 0.001 compared with CLS_8/9_CM + NG2 or NG2.

Finally, we investigated the proliferation potential of the cells within icMet cultures. As expected, after double IF staining for tyrosine phosphorylation (pTyr, red) in cMetβ (green)‐expressing MLpvNG2 cells (DAPI^+^), we detected more pTyr^+^/cMet(β)^+^ MLpvNG2 cells (DAPI^+^) when the cells in CLS_8/9_CM pretreated with icMet (icMet‐CLS_8/9_ + NG2, Figure 8F2, merged, arrows, 69.88% ± 12.01%) than the cells in the cultures without icMet (CLS_8/9_+NG2, Figure 8F1, merged, arrow, 28.87% ± 11.59%), and analysis of the observed difference was significant (Figure 8F3, *p* < 0.001), with percentages ~3 times greater in the cultures with icMet than in the cultures without icMet (Figure 8F4). These data confirmed that the prevention of apoptosis in MLpvNG2 cells occurs simultaneous to the stimulation of cell proliferation. These observations indicate that the Fas‐mediated apoptosis of MLpvNG2 cells in the LCM can be inhibited by blocking cMet signaling and that cell proliferation can be stimulated as well.

## 4. Discussion

Effective prevention or treatment of liver injury is a fundamental issue in the management of chronic hepatic diseases. Our previous studies have provided a proof of concept for the substantial effect of HGF signaling on stem cell therapy during the early stage of fibrosis [28]. However, for clinical use, cMet inhibition strategies that do not involve potentially harmful viral vectors need to be developed. In the present study, we inhibited the cMet receptor using a small peptide (icMet) at the CLS_8/9_ stage before and during donor cell therapy to observe the survival and fate of transplanted HSPs (MLpvNG2 cells) in the LCM after transplantation. This inhibitory approach promotes proliferation, prevents transplanted MLpvNG2 cells from undergoing Fas‐mediated apoptosis [19], and restores the biliary tree, particularly reconstructing sinusoidal structures to initiate hepatocyte regeneration probably by triggering host liver cell apoptosis [29] and reshaping the extracellular matrix (ECM) [27], thus leading to repopulation and 4‐week period long functional recovery of grafted MLpvNG2 cells.

Liver transplantation remains the last therapeutic option for life‐saving of end‐stage liver disease (ESLD), but a major obstacle is the shortage of available donor organs. Therefore, novel treatment options are needed to restore liver function. Although an obvious and broadly applicable option would be hepatocyte transplantation to restore the missing metabolic and synthetic capacity, the supply of donor organs is limited. Stem cells are an attractive source for bridging the limitation [30, 31]. However, regardless of the source, stem cells eventually die after transplantation through processes such as necroptosis, pyroptosis, ferroptosis [32], and cuproptosis [33, 34]. A widely accepted theory is that the majority of stem cells die after they are grafted into the injured liver. When they encounter pathological stresses beyond their maximal tolerance in the adverse milieu, transplanted stem cells may fail to survive and enter an irreversible cell death program [12, 13] rather than stably colonizing the tissue [35] and mediating therapeutic effects [36–38]. In our study, the transplanted MLpvNG2 cells may not have tolerated higher levels of HGF stress within LCM and could not stably colonize the harsh milieu; as such, apoptosis occurred. Therefore, stem cell‐based therapy for LC remains limited because of the massive loss of cells as apoptosis in the LCM after transplantation.

An ideal approach would be to develop strategies that prevent stem cell death or increase the rate of stem cell survival at cirrhotic sites to repopulate the diseased liver with functional cells differentiated from donor stem cells [9] and promote endogenous hepatic regeneration to maintain therapeutic effects. Several strategies have been used in recent decades to prevent apoptosis in the injured liver by targeting death effectors [39, 40] or inhibiting HGF signaling [41]. However, the underlying processes remain poorly understood. We addressed this issue by using well‐defined HSPs (MLpvNG2 cells) [7] as candidate therapeutic cells and well‐accepted DEN‐induced liver injury [8] or fibrosis/cirrhosis [7, 11] mouse models for MLpvNG2 cell transplantation, generating novel evidence.

First, we hypothesized that Fas‐mediated apoptosis was the predominant cause of the massive and rapid (18–24 h) loss of grafted cMet‐Fas‐expressing MLpvNG2 cells at the CLS_8/9_ stage after cell transplantation, and a strong burst of HGF was considered the main pathogenic stress in the milieu; the loss of MLpvNG2 cells was also confirmed when the cells responded to CLS_8/9_ liver cues (CLS_8/9_CM). Because cMet is known to be the only receptor for HGF [21], we also demonstrated that the suppression of HGF signaling by icMet led to the “rescue” of MLpvNG2 cells from apoptosis in the LCM and promoted long‐term healing. Finally, we elucidated the possible mechanism underlying the “rescued” MLpvNG2 cell‐mediated long‐term efficacy. It is possible to repopulate the diseased liver via direct differentiation of functional hepatic cells, such as chologiocytes (CK19) [7, 9]. Importantly, we specifically observed host LYVE‐1^+^ LSEC reconstruction in icMet‐pretreated DEN‐treated mice 4 weeks after MLpvNG2 transplantation, which indicated that the reconstruction supports regeneration of hepatocytes [24], such as ALB^+^ and G6Pc^+^ cells leading to long‐term therapeutic effects. Furthermore, cMet inhibition can relieve the fibrotic load (α‐SMA) [27], which may reduce HGF secretion [42] in the cirrhotic liver, promoting the association of the Fas–cMet complex association on MLpvNG2 cells to keep the cell survival [19]. In addition, we observed a substantial increase in cMetβ‐pTyr levels in MLpvNG2 cells (proliferation) when the cells were cultured in CLS_8/9_CM supplemented with icMet, which may also facilitate the replacement of MLpvNG2 cells lost due to apoptosis. By the way, as this study did not include an icMet‐only group, it was unable to distinguish if the protective effect of the combination therapy stemmed from a direct effect on the host liver or from a combined effect mediated by the protection of transplanted cells. The underlying mechanisms require further investigation. However, despite the limitation, combination therapy yields still significantly better outcomes than cell therapy alone.

In summary, the prior inhibition of cMet signaling by icMet during the cirrhotic stage before or during donor cell transplantation is a feasible strategy for optimizing stem cell‐based therapy. The present study results reveal that this novel strategy might be a critical approach for improving the poor retention of transplanted cells, such as hepatocytes, HSPs, and MSCs. Mechanistically, the lack of cMet signaling in the cirrhotic liver may remodel the diseased niche, which is beneficial for robust repopulation of transplanted stem cells [43], allowing the surviving donor cells (higher Ki‐67 and low TUNEL) to trigger a cascading reaction of liver regeneration not only participate directly in tissue repair via functional differentiation [44] but, more importantly, stimulate host cell regenerative potential (CK19, LYVE‐1, ALB, and G6Pc) through paracrine mechanisms, such as HGF secretion, or cell–cell interactions; and relieve the fibrotic load (α‐SMA), leading to long‐term efficacy, including the repair of the damaged liver (ALT and AST) and the promotion of anabolic (ALB) and excretive metabolism (TBiL and DBiL). Additionally, the icMet reagent, which does not involve potentially harmful viral vectors, is an important tool for future clinical use.

## Author Contributions

Min Yan performed most of the experiments, prepared the figures, and analyzed some of the data. Deyu Hu, Jiayu Yu, Quanyu Chen, and Jiejuan Lai performed staining and RT‐qPCR and analyzed some of the experimental data. Mei Ding and Lu Li analyzed some of the data. Hongbo Shi obtained financial support. Hongyu Zhang and Yu Chen guided the analysis of the data and obtained financial support. Lianhua Bai contributed critically to the analysis of all the data, assisted with the interpretation of the results, designed all the experimental procedures, and finalized the manuscript.

## Funding

This study was supported by grants from the following agencies: the National Natural Science Foundation of China (Grants 81873586 and 81571566); the Capital Medical University Beijing You’an Hospital Youth Innovation Project (Grant 310315301); and the National Key Research and Development Program of China (Grant 2022YFC2304402).

## Conflicts of Interest

The authors declare no conflicts of interest.

## Supporting Information

Additional supporting information can be found online in the Supporting Information section.

## Supporting information


**Supporting Information** The following are the Supporting Information, including two tables (Table S1 and Table S2) and two figures (Figure S1 and Figure S2). Table S1: The sequences of seven primers used to detect mRNA expression in the livers of C57BL/6 mice from different groups by qRT‐PCR. Table S2: This table lists all of the biological materials used in this study, including antibodies (primary, secondary), reagents, kits, media and buffers, with names, catalog numbers, applications, companies, cities and countries. Figure S1: Images of pathological processes evaluated by established DEN‐induced liver fibrosis/cirrhosis mouse modelmouse model generated by administering C57BL/6 mice with DEN through drinking water; the donor cell transplantation procedure and endogenous hepatic stem/progenitor cell survival status during different periods of DEN‐induced liver injury were determined on the basis of our previous studies. Figure S2: The survival pattern of human intrahepatic NG2‐expressing cells (hNG2) in nonspecific inflammatory hemangioma (HA) livers and cirrhotic livers, was almost similar to that of mouse intrahepatic NG2‐expressing cells (eNG2 [11]).

## Data Availability

The data that support the findings of this study are available from the corresponding author upon reasonable request.
