# Supplementary material for: A cMet Inhibitor Promotes the Survival and Long‐Term Efficacy of Transplanted Hepatic Stem/Progenitor Cells in Liver Cirrhosis
Source: Stem Cells Int. 2026 Oct 30;2026:7020341. doi: 10.1155/sci/7020341 (PMC13720526; doi:10.1155/sci/7020341)
Supplement: Supplementary file 1 [file SCI-2026-7020341-s001.docx]

**SUPPLEMENTARY MATERIALS**

This supplementary information includes two supplementary tables (Table S1, Table S2) and two supplementary figures (Figure S1, Figure S2) .

**Table S1** This table enumerates the seven primer sequences used for detecting mRNA expression in C57BL/6 mouse livers from different groups by qRT-PCR assay.

**Table S2** This table centrally lists all the biological items used in this study, including Antibodies (primary, secondary), Reagents, Kits, Mediums and Buffers, with item Names, Catalogue numbers, Applications, Companies, Cities and Countries.

**Figure S1** This Figure provided images of pathological processes in DEN-induced liver fibrosis/cirrhosis mouse model through diet DEN in drinking water in C57BL/6 mice determined by certain biological methods; donor cell transplant plan and endogenous hepatic stem/progenitor cell survival situation in different periods of the DEN-injured livers based on our previous similar studies.

Study design: **(A)** Masson's Trichrome staining (blue) marks the pathological process from naive to fibrosis (3-6 weeks post-DEN) to cirrhosis (6-10 weeks post-DEN) and to cancer (>10 weeks post-DEN), pink arrow indicates appearance of cancer. **(B)** Diagram of MLpvNG2 cell transplant plan into the DEN model mice with or without icMet (blocking cMet receptor) administration; MLpvNG2 cell purification procedure was previously reported. Groups: the MLpvNG2 cells were labeled with CellDye (either CFSE or RedDye) and injected IV into the DEN-mice fibrotic (FLS_3/4_) and cirrhotic (CLS_8/9_) livers respectively, and analyzed at 3 days (assumed short-term therapy) or 4 weeks (assumed long-term therapy) after the MLpvNG2 cell transplantation. **(C)** Immunufluorescence (IF) staining (red) for neuro-glia antigen 2 (NG2) glycanprotein expressing cells in fibrotic (3-4 weeks post-DEN) and cirrhotic (8-9 weeks post-DEN), arrows indicate NG2^+^ cells. **(D)** Double IF staining for Ki-67 **(**green**)** and NG2 (red) for detecting proliferation of intrahepatic NG2^+^ cells (eNG2) in the DEN-mouse livers, arrows indicate merged Ki-67^+^ cells in NG2^+^ cells. **(E)** Double IF staining for TUNEL **(**green**)** and NG2 (red) for detecting apoptotic eNG2^+^ cells in the model injury livers, arrows indicate merged TUNEL^+^ cells in eNG2^+^ cells. Image size: Scale bar=200 μm. Animal sample size: the number of animals per group was determined and analyzed based on previous similar studies.

**Figure S2** This Figure provided similar survival evidence of human intrahepatic NG2-expression cells (hNG2) to mouse intrahepatic NG2-expression cells (eNG2[2]) in inflammatory hemangioma livers and HBV/cirrhotic livers.

Study design: **(A)** IF staining (red) marks eNG2^+^ cells in human liver samples (hNG2) with hemangioma (HA as controls); **(B)** IF staining (red) marks NG2^+^ cells in human liver samples with hepatic B virus (HBV)/cirrhosis (hcirLiver, box); boxes indicate hNG2^+^ cell regions. Double IF staining for comparative expression of TUNEL in hNG2^+^ cells between HA **(A**, merged**)** and hcirLivers **(B**, merged**)**, arrows indicate hNG2^+^ cells stained by TUNEL. **(C)** Quantification analysis for hNG2^+^ cells by IF staining (red) in HA (A) and hcirLivers (B). **(D)** Quantification analysis for TUNEL^+^/hNG2^+^ cells by double IF staining (merged) in HA (A) and hcirLivers (B). Sample size: The experimental human samples were with 3 in HA and **3** in hcirLiver. Blinding: Two distinct researchers were involved in the human sample testing: One for the collecting samples, and subsequently staining, while the other for the data analysis. Image size: Scale bar=200 μm. Outcome measures: The following parameters were evaluated: Endogenous hNG2-expressing (red, boxes) andapoptotic (merged, arrows) status in human HA and hcirLiver samples. Statistical methods: All kinds of staining in this study were performed in triplicate or more and the presented data are presented as the means±SDs, ***p*<0.001 compared hcirLiver samples with the HA samples.

**Figure S1** **DEN-induced chronic liver injury model mice from naive to fibrosis to cirrhotic to cancer for extrahepatic MLpvNG2 cell transplantation and intrahepatic activity testing of the cells.**

**Figure S2 Survival fate of NG2-expressing cells (hNG2) in human HBV/cirrhotic (hcirLiver) and hemangioma (HA) liver samples.**

**Table S1. Primer sequences for qRT-PCR**

| **Genes** | **Forward Primers** | **Reverse Primers** |
| --- | --- | --- |
| *hgf* | ACTTCTGCCGGTCCTGTTG | CCCCTGTTCCTGATACACCT |
| *g6pc* | CGACTCGCTATCTCCAAGTGA | GTTGAACCAGTCTCCGACCA |
| *alb* | TGCTTTTTCCAGGGGTGTGTT | TTACTTCCTGCACTAATTTGGCA |
| *acta2* | AAAAGACAGCTACGTGGGTGA | GCCATGTTCTATCGGGTACTTC |
| *krt19* | GGGGGTTCAGTACGCATTGG | GAGGACGAGGTCACGAAGC |
| *lyve1* | CAGCACACTAGCCTGGTGTTA | CGCCCATGATTCTGCATGTAGA |
| *actb* | GGCTGTATTCCCCTCCATCG | CCAGTTGGTAACAATGCCATGT |

***hgf***, hepatocyte growth factor; ***g6pc***, glucose-6-phosphatase catalytic; ***alb***, albumin; ***acta2***, actin alpha 2; ***krt19***, keratin 19; ***lyve1***, lymphatic vessel endothelial hyaluronan receptor 1; ***actb***, actin beta

**Table S2. Experimental biological iteams**

| **Items** | **Name(s)** | **Cat. No.** | **Application(s)** | **Companies** | **Cities, States** | **Countries** |
| --- | --- | --- | --- | --- | --- | --- |
| **Antibodies**  **(Abs)** |  |  |  |  |  |  |
| **Primary Ab**s | Anti-HGF | 115353 | Immunofluorescence staining, western blot analysis | Invitrogen | California | USA |
|  | Anti-FADD | 14906 |  | Proteintech | Wuhan | China |
|  | Anti-caspase-8 | 66093 |  | Proteintech | Wuhan | China |
|  | Anti-caspase-3 | 66470 |  | Proteintech | Wuhan | China |
|  | Anti-Fas | sc-74540 |  | Santa Cruz Biotechnology | California | USA |
|  | Anti-cMet (Co-IP) | 25H2 |  | CST | Danvers, Massachusetts | USA |
|  | Anti-Ki-67 | bs-2130R |  | Bioss | Beijing | China |
|  | Anti-albumin (ALB) | 66051 |  | Proteintech | Wuhan | China |
|  | Anti-glucose-6-phosphatase (G6PC) | bs-21523R |  | Bioss | Beijing | China |
|  | Anti-CK19 | 14965 |  | Proteintech | Wuhan | China |
|  | Anti-ɑ-SMA | 23081 |  | Proteintech | Wuhan | China |
|  | Anti-LYVE-1 | 14-0443-80 |  | Invitrogen | California | USA |
|  | Anti-phosphorylated tyrosine (pTyr) | ET1704-20 |  | HUABIO | Hangzhou | China |
|  | Anti-NG2 | AB5320B |  | Millipore | Massachusetts | USA |
|  | Anti-CD90 | 66766 |  | Proteintech | Wuhan | China |
| **Secondary Abs** | Alexa Fluor 488-conjugated AffiniPure goat anti-mouse IgG (H+L) | 115-545-003 | Immunofluorescence | Jackson ImmunoResearch | Pennsylvania | USA |
|  | Alexa Fluor 488-conjugated AffiniPure goat anti-rabbit IgG (H+L) | 111-545-003 |  |  |  |  |
|  | Cy3-conjugated AffiniPure goat anti-mouse IgG (H+L) | 115-165-003 |  |  |  |  |
|  | Cy3-conjugated AffiniPure goat anti-rabbit IgG (H+L) | 111-165-003 |  |  |  |  |
| **Reagents** | icMet | HY-15735 | a cMet receptor inhibitor | MCE | New Jersey | USA |
|  | FBS | 10270 | Cell cultures | Gibco | California | USA |
|  | Penicillin/streptomycin | C0222 | Cell cultures | Beyotime | Shanghai | China |
|  | Tyrode’s salt solution | T2145 | Cell cultures | Sigma‒Aldrich | St. Louis, Missouri | USA |
|  | DEN | NO756 | Animal models | Sigma‒Aldrich | St. Louis, Missouri | USA |
|  | Carboxyfluorescein diacetate succinimidyl ester (CFSE)  (rHGF) | 1948067 | Cell tracking | Invitrogen | California | USA |
|  | Red CM–DiI | C7000 |  | Invitrogen | California | USA |
|  | Percoll | 2218810 | Cell culture | Langtian Biotechnology | Chongqing | China |
|  | 4'-6-Diamidino-2-phenylindole (DAPI) | C1006 | IF | Beyotime | Shanghai | China |
|  | DAB | ZL1-9018 | H&E | ZSGB-Bio | Beijing | China |
|  | TRIzol | 9109 | RT‒qPCR | TaKaRa | Shiga | Japan |
|  | Protease inhibitors | P1050-1 | WB, Co-IP | Beyotime | Shanghai | China |
|  | GammaBind Plus Sepharose | 17088602 | Co-IP | GE Healthcare | Logan, UT | USA |
| **Kits** | BCA protein assay | P0010S | WB, Co-IP | Beyotime | Shanghai | China |
|  | TUNEL assay kit | C1090 | IF | Beyotime | Shanghai | China |
|  | Boyden chambers |  |  | Millipore | Billerica, MA | USA |
|  | Reverse transcriptase polymerase chain reaction kit | RR820A | qRT‒PCR | TaKaRa | Shiga | Japan |
| **Mediums** | Eagle’s medium without calcium and magnesium ions (_W_MEM) | SH30346.03 | Cell culture | GE Healthcare | Logan, UT | USA |
|  | DMEM/F-12 | 6123189 |  | Gibco | Paislay | UK |
| **Buffers** | Lysis buffer | P0013 | Co-IP | Beyotime Biotechnology | Shanghai | China |
